# Editorial: Multipurpose prevention technologies for HIV, STIs and pregnancies

**DOI:** 10.3389/frph.2024.1384153

**Published:** 2024-02-27

**Authors:** Barbara A. Friedland, Andrea Ries Thurman, Harriet Nuwagaba-Biribonwoha, R. Karl Malcolm

**Affiliations:** ^1^ Center for Biomedical Research, Population Council, New York, NY, United States; ^2^ Dare Bioscience, San Diego, CA, United States; ^3^ Eswatini Prevention Center Clinical Research Site, Mbabane, Eswatini; ^4^ Department of Epidemiology, Mailman School of Public Health, Columbia University, New York, NY, United States; ^5^ School of Pharmacy, Queen’s University Belfast, Belfast, United Kingdom

**Keywords:** multipurpose prevention technologies (MPTs), reproductive health, HIV/AIDS, sexually transmitted infections (STIs), family planning (FP), contraception, pre-exposure prophylaxis (PrEP)

**Editorial on the Research Topic** Multipurpose prevention technologies for HIV, STIs and pregnancies

**Figure 1:**
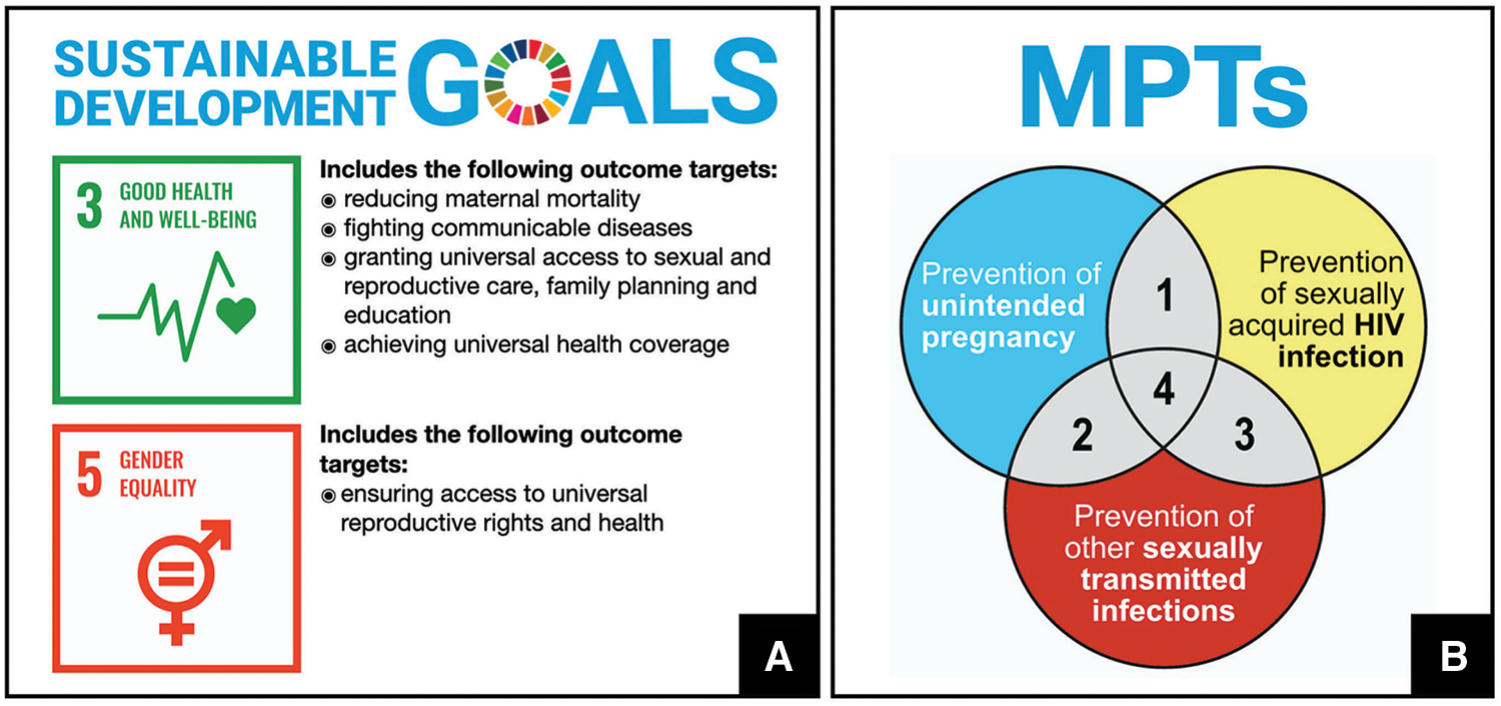
(**A**) Sustainable Development Goals aimed at improving the health and well-being of women. (**B**) Multipurpose prevention technology (MPT) products are biomedical products intended to address three inter-related sexual and reproductive health issues simultaneously: prevention of unintended pregnancy, prevention of sexually transmitted infections, prevention of sexually-acquired infection with human immunodeficiency virus (HIV).

Women worldwide face three overlapping risks that significantly impact their health and well-being: HIV/AIDS, sexually transmitted infections (STIs), and unintended pregnancy. In 2022, more than half of the approximately 38 million people living with HIV were women and girls (1). According to the WHO, over one million STIs are acquired every day worldwide (2) often leading to lifelong complications for females, such as infertility and chronic pelvic pain (3). Half of pregnancies each year are unintended with over 60% ending in abortion (4). These statistics underscore gender inequalities that disproportionately affect poor women with lower levels of education and limited access to modern healthcare (5). Two of the 17 Sustainable Development Goals (SDGs)—Goals 3 and 5 (Figure 1A)—are aimed at improving women's sexual and reproductive health (SRH) (6).

Multipurpose prevention technologies (MPTs) are emerging biomedical interventions to prevent two or more SRH issues simultaneously (Figure 1B) (7, 8). Male and female condoms, the only existing MPTs, have drawbacks limiting their consistent use, particularly among the most vulnerable (9–11). Novel MPTs often integrate drug delivery and medical device functions within a single product to increase adherence and overall effectiveness (7). This special issue includes 12 articles from leading researchers, healthcare providers, policymakers and program managers describing recent advances and considerations for the development, scale-up and introduction of MPTs.

Five articles feature MPTs containing the antiretroviral ***tenofovir*** (TFV), used (with emtricitabine) for HIV pre-exposure prophylaxis (PrEP) (12). Two articles focus on the ***dual prevention pill (DPP)*** for HIV and pregnancy prevention—a daily oral tablet containing tenofovir disoproxil fumarate (TDF) and emtricitabine, and an ethinyl estradiol (EE)/levonorgestrel (LNG) combined oral contraceptive. The DPP is the MPT furthest along in development, with an estimated FDA filing in 2025 (13). Segal et al. recommend DPP counselling guidelines developed by a working group to address the different labels for PrEP and oral contraceptives, including if women could safety “double up” or skip the last week of a DPP pack (the “placebo period”) in alignment with the oral contraceptive regimen. Milali et al. present cost-effectiveness modelling of the DPP for different populations (e.g., women in sero-discordant relationships, sex workers, general population) in Kenya, South Africa and Zimbabwe. The authors conclude that the DPP could be cost-effective and even cost-saving in populations at substantial HIV risk, but that outcomes will be sensitive to adherence, underscoring the importance of effective counselling.

Patel et al. describe pre-clinical research on 20 and 40 mg TFV doses in a ***quick-dissolving polymeric thin film*** to prevent HIV and herpes simplex virus (HSV). Results of stability, *ex vivo* HIV-1 challenge experiments, and safety assessments (tissue, microbiome, neutrophil influx, and pH) in Rhesus macaques indicate that the films were stable, safe, and efficiently delivered TFV. Two articles report on clinical trials of a vaginal ring combining TFV and LNG for HIV, HSV and pregnancy prevention. Mugo et al. demonstrate that the 90-day ***TFV/LNG ring*** is acceptable, safe, and well tolerated among Kenyan women using it for up to 90 days in a Phase IIa trial, and Tolley et al. report from a Phase I trial that the ring is acceptable among women in the Dominican Republic and the United States. Tolley et al. note that modifications to decrease the ring's size/thickness and extend its use period could further increase acceptability and emphasize the need to develop communication strategies to demystify ring use for women who are naïve to vaginal product use.

Shapley-Quinn et al. present qualitative acceptability findings from a Phase I trial of a vaginal ring that combines LNG with the antiretroviral ***dapivirine*** (DPV) for HIV and pregnancy prevention. The dapivirine ring (DVR) was the first approved vaginal microbicide and is currently being introduced in multiple African countries (14–19). The ***DPV-LNG ring,*** being developed as a line-extension of the DVR, was well-tolerated in a Phase I trial (20), with overwhelming support for a 90-day product. However, most participants felt that their personal risk of HIV infection or motivation to use the product for contraception did not outweigh their experiences of partial/complete expulsions or increased incidence of vaginal bleeding. Participants’ feedback was critical for informing an updated DPV-LNG ring design being tested in a Phase I trial (21), emphasizing the importance of including qualitative research early in product development.

Gachigua et al. describe a human-centered design study assessing the potential acceptability, usability, and programmatic fit of a drug-eluting microarray patch (MAP) in Kenya. MAPs administer drugs through the skin using an array of tiny needles (22–24). Through focus group discussions with various end-user groups, mock exercises in which participants tried prototype MAPS, and key informant interviews, the authors conclude that MAPs are acceptable for both HIV prevention and as an MPT.

Five papers in this special issue discuss overall considerations or provide recommendations for ongoing MPT development. Bhushan et al. share their novel conceptual model for use in developing and testing MPT acceptability. The model, developed in the context of a scoping review of previously conducted end-user research, builds on previous conceptual models and incorporates influencing factors (individual, partner, provider, community) with MPT acceptability factors (including overall acceptability and relative acceptability to other products) as drivers of MPT preference, adoption and use.

Holt et al. describe the current MPT landscape and propose strategic actions for MPT development and introduction in low- and middle-income (LMIC) countries. Based on insights from 28 key informants (e.g., product developers, regulatory experts, policymakers, community stakeholders) from multiple regions, the authors provide recommendations in six areas: technical challenges and opportunities; regulatory pathways; advancing from pre-clinical to clinical development; cost and market potential; market access; and product introduction and roll-out. A commentary by Dam et al. contains insights from the donor agency perspective highlighting three factors requiring global, regional, and local stakeholder coordination to successfully introduce and scale-up MPTs: (i) procurement and supply chain barriers; (ii) potential burden on health systems; and (iii) impact on current programs.

Two articles call for expanding MPT development beyond the current products that focus primarily on HIV and unintended pregnancy. Lu and Haddad encourage more research on products to prevent non-HIV STIs, outlining a strategy that includes harnessing the large potential market for non-HIV STI prevention in developed countries that could engage investors who have not yet partnered with MPT developers. Finally, Behrsteyn et al. urge developers to consider an array of products for women at various points in their lives including pre-conception, pregnancy, lactation, and menopause (25). Specific product combinations could include prenatal supplements with HIV and STI prevention, emergency contraception with HIV post-exposure prophylaxis, or hormone replacement therapies for menopause with HIV/STI prevention.

The breadth of choice offered by the various MPTs in development—similar to existing options for contraception—is encouraging and critical to empowering women to make important SRH decisions (26). While MPTs hold great promise, there are many challenges—scientific and technical, regulatory and approval, user acceptance and adherence, funding and resource allocation, marketing and distribution, ethical and equity considerations, education and awareness, and integration into current health systems. These challenges will require a multidisciplinary approach involving researchers, healthcare professionals, policymakers, and community stakeholders to ensure MPTs fulfil their potential in improving women's SRH outcomes.
